# Supplementary material for: What role can videogames play in the COVID-19 pandemic?
Source: Emerald Open Res. 2020 Jun 2;2:34. doi: 10.35241/emeraldopenres.13727.1 (PMC7453828; doi:10.35241/emeraldopenres.13727.1)
Supplement: Peer review history

# Peer review: Reviewer response for version 1

**DOI:** 10.21956/emeraldopenres.14802.r27060

**Published:** 2020-08-26

**Authors:** Frans Mäyrä

**Affiliations:** 1 Faculty of Information Technology and Communication Sciences, Tampere University, Tampere, Finland

This is an interesting and well-studied article that brings together a lot of research from game studies and the games-for-health field in particular. It is good to note that health and well-being is here approached from a wide-ranging and inclusive perspective, taking sociocultural aspects of health also into consideration. The role of loneliness and social connectedness is particularly emphasised, which is a relevant choice, considering the role of games and play as particular kinds of popular social activities. There is also a topical framing of this article in the recent COVID-19 pandemic and its associated risks for social isolation and loneliness. 

 The main body of research literature and discussion in this short article is directed towards addressing games’ potential for positively supporting health, particularly in the case of PTSD (post-traumatic stress disorder). The suggestion of authors is that under the pandemic, health workers would particularly benefit from the use of games for recovery. This seems plausible, and also supported by literature (though, it should be noted that I am not an expert in health or therapies). What might be missing, or not given similar kind of treatment, is the games’ relationship to the socially isolating and stressful conditions in everyday lives of ”non-professionals” (i.e. in lives of families, students, and many other people). But, as said, this is a short article and a certain focus is a good thing.

 From a game studies perspective, it should also be noted that there is not much discussion about the differences between different genres or forms of games and play that actually vary a lot. Games are discussed in very overarching and generalising terms. There are also significant differences in players’ responses of games, and while one kind of game might be relaxing and therapeutic for some, it might be frustrating and anxiety creating for someone else. Such, more fine-grained discussions that are relevant to game studies have fallen outside of the scope of this short article, but it would be welcome to see at least some short references into such directions.

 The article is logically structured and generally well-written, but there were some instances of word choices and sentence structures that left me wishing for yet one more round of minor stylistic proof-reading. Maybe such small amendments could still be made into the text? Otherwise I think that this article is solid work, puts forward a valuable perspective and contribution, and should indeed be indexed.

Is the topic of the opinion article discussed accurately in the context of the current literature?

Yes

Are arguments sufficiently supported by evidence from the published literature?

Yes

Are all factual statements correct and adequately supported by citations?

Yes

Are the conclusions drawn balanced and justified on the basis of the presented arguments?

Yes

Reviewer Expertise:

Game studies, game culture studies, humanities, art and culture studies.

I confirm that I have read this submission and believe that I have an appropriate level of expertise to confirm that it is of an acceptable scientific standard.

# Peer review: Reviewer response for version 1

**DOI:** 10.21956/emeraldopenres.14802.r26958

**Published:** 2020-07-16

**Authors:** Dorothy Newbury-Birch

**Affiliations:** 1 School of Social Sciences Humanities and Law, Teesside University, Middlesbrough, UK

This is a very good piece of work that is worthy of publication. I only have a couple of small things to consider:

 On page 3 when talking about COVID-19 I suggest that papers relating to COVID-19 are referenced separately to other data that says the same thing.

 I think there should be some mention of testing through proper RCTS the effectiveness and cost-effectiveness of interventions.  The Lyadurai et al study had a one week follow up and the paper indicates that it would be better at a month however that is not a long enough time to really  consider whether it is effective.

 Well done to the authors - this is a well written article.

Is the topic of the opinion article discussed accurately in the context of the current literature?

Yes

Are arguments sufficiently supported by evidence from the published literature?

Yes

Are all factual statements correct and adequately supported by citations?

Yes

Are the conclusions drawn balanced and justified on the basis of the presented arguments?

Yes

Reviewer Expertise:

Public Health

I confirm that I have read this submission and believe that I have an appropriate level of expertise to confirm that it is of an acceptable scientific standard.
